# CARving the Path to Allogeneic CAR T Cell Therapy in Acute Myeloid Leukemia

**DOI:** 10.3389/fonc.2021.800110

**Published:** 2022-01-10

**Authors:** Oren Pasvolsky, May Daher, Gheath Alatrash, David Marin, Naval Daver, Farhad Ravandi, Katy Rezvani, Elizabeth Shpall, Partow Kebriaei

**Affiliations:** ^1^ Department of Stem Cell Transplantation and Cellular Therapy, University of Texas M.D. Anderson Cancer Center, Houston, TX, United States; ^2^ Institute of Hematology, Davidoff Cancer Center, Rabin Medical Center, Petah-Tikva, Israel; ^3^ Sackler Faculty of Medicine, Tel Aviv University, Tel Aviv, Israel; ^4^ Department of Leukemia, University of Texas MD Anderson Cancer Center, Houston, TX, United States

**Keywords:** immune effector cell therapy, acute myeloid leukemia, cancer immunotherapy, chimeric antigen receptor T cells, allogeneic

## Abstract

Despite advances in the understanding of the genetic landscape of acute myeloid leukemia (AML) and the addition of targeted biological and epigenetic therapies to the available armamentarium, achieving long-term disease-free survival remains an unmet need. Building on growing knowledge of the interactions between leukemic cells and their bone marrow microenvironment, strategies to battle AML by immunotherapy are under investigation. In the current review we describe the advances in immunotherapy for AML, with a focus on chimeric antigen receptor (CAR) T cell therapy. CARs constitute powerful immunologic modalities, with proven clinical success in B-Cell malignancies. We discuss the challenges and possible solutions for CAR T cell therapy development in AML, and examine the path currently being paved by preclinical and clinical efforts, from autologous to allogeneic products.

## Introduction

### Acute Myeloid Leukemia—The Unmet Need

Traditional intensive chemotherapy regimens for AML have generated long term survival rates of up to 50% in younger patients, and lower than 40% in patients over the age of 60 (1, 2). Allogeneic stem cell transplant (alloHCT) can further reduce risk of relapse. A meta-analysis revealed improved overall survival (OS) as well as relapse free survival (RFS) for patients with intermediate or poor risk disease who undergo alloHCT in first complete remission (CR1) (3). Yet non relapse mortality (NRM) remains non-negligible, and many patients still relapse (4).

In recent years we have witnessed an influx of revolutionary strategies in AML treatment. Advances in the understanding of the molecular biology of AML have enabled identification of genetic mutations which confer prognostic significance as well as susceptibility to chemotherapy (5). Biological agents targeting specific mutations have been incorporated into the armamentarium against AML. Notably, addition of midostaurin to induction chemotherapy resulted in a 22% reduction in the risk of death compared to placebo in patients younger than 60 years of age with fms-related tyrosine kinase 3 gene (FLT3) mutated AML (6). Promising results have been observed with the addition of isocitrate dehydrogenase (IDH) inhibitors. The addition of ivosidenib or enasidenib to induction chemotherapy yielded an estimated 1-year survival rate of 78% and 76% in patients with either IDH1 or IDH2 mutations, respectively (7).

Despite therapeutic advances, long term remission rates and survival for AML patients remain suboptimal, with improvement in survival throughout the years observed mainly in the younger population (8). In the elderly patients and those with comorbidities, intensified treatment has usually overlapped with increased toxicity, preventing optimal treatment (9). The advent of hypomethylating agents (HMA), especially in combination with the BCL2 inhibitor venetoclax, has yielded relatively high remission rates and prolonged survival with a favourable toxicity profile in patients deemed unfit for intensive chemotherapy (10). Nonetheless, remissions achieved are usually short lived, and relapsed AML remains extremely challenging.

## Immunotherapy in AML

### The Rationale

There is growing understanding of the importance of the BM microenvironment for AML survival and progression. Various cells in the BM niche, including immune mediators, play a crucial part in this interaction. Bone marrow CD8+ T cells in patients with AML have been shown to exhibit an exhausted phenotype and to have upregulation of the inhibitory molecule PD1 (11). Upregulation of the TIM-3/Gal-9 axis, also believed to play a role in T cell exhaustion, has been observed in patients with refractory AML (12). Therefore, microenvironment-targeted treatments are being developed. For instance, CXCR4 expression has been shown to be upregulated after chemotherapy, providing a mechanism of therapeutic resistance in pediatric AML (13). CXCR4 inhibitors have been incorporated into different treatment regimens in acute leukemias. Phase I studies have shown feasibility of adding the CXCR4 antagonist Plerixafor to sorafenib and GCSF in FLT3-mutated R/R AML (14), or to high-dose cytarabine and etoposide in pediatric patients with acute leukemias or myelodysplastic syndrome (MDS) (15).

Another example is the chemoresistance of leukemic blasts conferred by hypoxia. In an animal model, AML progression was facilitated by hypoxic microenvironment (16). PR104, a prodrug that converts to an active DNA cross linker in hypoxic conditions, has been assessed in a phase I/II clinical trial for patients with R/R ALL or AML. Of the 31 AML patients receiving the higher dose of PR104, 32% responded, yet substantial hematologic and non-hematologic toxicity was reported (17).

The immune landscape in different malignancies influences the potential efficacy of immunotherapy. This includes variability in the presence of cytotoxic lymphocytes, HLA II expression, activating and inhibitory signals produced by tumor cells and antigen presenting cells (APCs), as well as cancer neoantigens. In a broad analysis of patients with hematological malignancies, those with acute leukemias had the lowest cytolytic scores, reflecting lower T and NK cell fractions in the bone marrow (18). Certain AML subgroups appear to have distinct immunological phenotypes. For instance, patients with an MDS-like transcriptome had increased bone marrow cytolytic infiltration. Bone marrow and peripheral blood CD3 and CD8 levels have been show to predict response to PD1 inhibition combined with hypomethylating therapy in patients with R/R AML (19).

Single cell level CD4+ and CD8+ cell functional proteomics analysis of patients receiving the aforementioned PD1 inhibitor-based immunotherapy revealed that the pre-treatment bone marrow CD4+, but not CD8+, functional signature correlated with clinical responses, mainly driven by expression of IFNγ and TNFα (20). Several factors in the AML microenvironment might hamper success of CAR T cells. For instance, an increase in regulatory T cells (Tregs) has been observed in AML (21). Activation and proliferation of cytolytic T cells are hindered by Tregs, and depletion of endogenous Tregs has been shown to improve the efficacy of adoptive cytotoxic T-cell immunotherapy in a mouse model of AML (22). Therefore, blocking Treg effect by disruption of the IL2 axis has been achieved by genetic alteration of the PYAP Lck-binding motif of CD28 costimulatory domain, when combined with 4-1BB CAR T costimulatory signalling in solid tumors (23).

Bruk et al. compared immune profiles of AML patients to those of chronic myeloid leukemia (CML), B-cell acute lymphoblastic leukemia (B-ALL), as well as a group of controls (24). Overall, the expression of immune checkpoint receptors in the bone marrow of AML patients was low, however authors discovered two age dependent immunologic signatures which differed by TCR clonality and clinical prognosis. An analysis of 442 bone marrow samples from three pediatric and adult AML cohorts has further elucidated the correlation between the immune landscape and response to immunotherapy (25). Authors performed transcriptional microenvironment stratification into immune subtypes, which predicted differential responses to immunotherapy. Tumor-intrinsic factors, including mutations in cancer driver or tumor suppressor genes, correlated with the heterogeneity of immune infiltration. For instance, patients with TP53 mutated AML had an IFNγ-predominant immune microenvironment, suggesting potential benefit for immunotherapy in this subgroup with poor prognosis.

### Antibody-Based Therapies

Monoclonal antibodies (Abs) have been utilized to target specific cell markers expressed on AML cells. These can be naked antibodies or conjugated to cytotoxic agents. Gemtuzumab ozogamicin (GO) is humanized anti-CD33 monoclonal Ab conjugated with calicheamicin. Despite initial safety concerns, later studies with adjusted GO regimens confirmed survival benefit and acceptable toxicity, especially in patients with non-poor risk AML (26). IMGN632, an anti-CD123 monoclonal Ab conjugated to a novel DNA alkylating agent, has shown some efficacy in patients with relapsed/refractory (R/R) AML or blastic plasmacytoid dendritic cell neoplasm (BPDCN). Forty-three percent of the 66 patients in the AML cohort achieved an objective response (of whom 3 CR). Severe neutropenia was the most common adverse event, and veno-occlusive disease (VOD) was noted in 2 patients (27).

Bispecific T-cell engagers (BITES)/bispecific killer cell engagers (BIKES): These products link unmanipulated endogenous effector T or NK cells to tumor-expressed surface antigens, activating the cytotoxic potential of a patient’s own T cells to eliminate cancer cells. These molecules include two binding domains, which are single-chain variable fragment (scFv) regions from monoclonal antibodies. The one scFv domain recognizes a tumour expressed antigen and the second recognizes the effector cell. One example for BITE use in AML is AMG 330, where the N-terminal domain of the drug recognizes CD33 and the C-terminal domain recognizes CD3ϵ on T cells. Delivery requires continuous intravenous infusion, and like other immunologic therapy, a common adverse event noted in 67% (including 13% with grade 3 or above) of the 55 patients with R/R AML enrolled in the phase 1 study of AMG 330 was cytokine release syndrome (CRS). CRS was reversible and occurred in a dose/schedule-dependent manner. Across the 16 cohorts, with 4 scheduling options of dose escalation, 19% of evaluable participants responded, including 3 CRs (one with negative minimal residual disease (MRD) (28).

Using separate scFv sets for two different targets in one molecule has also been developed. This bivalency enables the drug to distinguish different cell types, therefore increasing on-target specificity. AMV564 is a bivalent, bispecific (2:2) T cell engager that binds CD33 and CD3. Phase 1 data in 30 R/R AML patients revealed limited toxicity, with no grade 3 or higher CRS, yet 9 patients (30%) had febrile neutropenia grade 3 or above. In 17 (63%) of evaluable patients marrow blast reductions were observed (29).

Dual affinity retargeting antibodies (DARTs) – These molecules offer enhanced binding efficiency compared to BITES. This is achieved by manufacturing two Fv fragments, with swapping of the VH portion between the two fragments, providing binding properties more similar to those of the natural IgG molecule. Whereas BITES are composed of a single polypeptide with one N- and one C-terminus, DARTs contain two separate polypeptide chains bounded noncovalently in the diabody format, as well as additional stabilization through a C-terminal disulfide bridge (30). A phase 1/2 study with Flotetuzumab, a CD123/CD3ϵ directed DART administered by continuous IV infusion, enrolled a total of 88 adults with relapsed/refractory AML (31). Infusion-related reaction (IRR)/CRS was reported in all patients, yet most were grade 2 or lower, and none grade 4/5. Notably, a particularly high-risk group of 30% patients refractory to induction chemotherapy or with early relapse had a relatively high overall response rate of 30%,

Further enhancing the DART structure, SPM2 has been developed as a tri-specific agent with dual binding sites for the leukemic antigens CD33 and CD123, as well as for the effector NK cell marker CD16. *In vitro* studies on human AML cells show promising activity. Of note, leukemic stem cells showed increased susceptibility to SPM-2, presumably due to increased combined antigen density (32).

### Tumor Vaccines

Anti-cancer vaccines are designed to enhance presentation of tumor specific antigens in order to disrupt tolerance and augment T-cell immunity. In AML, vaccines have been utilized mostly in patients with a low-burden of disease, since they are believed to have better immune responses in that setting. Wilms’ tumor 1 (WT1) has been one of the most widely explored vaccine targets in AML, since it is a leukemia-associated protein overexpressed in leukemic cells with a pivotal role in blast proliferation and differentiation (33). In a phase II trial, 22 AML patients in CR1 received up to twelve doses of a heteroclitic WT1 peptide vaccine, designed to stimulate responses from both CD8 as well as CD4 lymphocyte subsets (34). The treatment was well tolerated, and in most patients a CD4/CD8 immune response was elicited. Median disease free survival was 16.9 months, and interestingly, none of the patients who developed a CD4 immune response relapsed. A novel approach has employed enhanced antigen presentation using allogeneic dendritic cells in a multi-antigen-expressing tumor cell vaccine. Preliminary results of a phase II trial in ten MRD positive AML patients have been reported (35). The treatment was safe, and two patients converted to MRD negativity lasting at least one year.

### Engineered T-Cell Receptor Therapy

Similar to the concept of CAR T-cells described in the following section, T-cell receptor (TCR) T-cell therapy also utilizes ex vivo T-cell modification in order to battle tumor cells. However, TCR T-cells use a distinct antigen recognition mechanism. Whereas TCRs use alpha and beta peptide chain heterodimers to recognize polypeptide fragments presented by MHC molecules, CAR Ts use antibody fragments that bind to antigens on the surface of malignant cells (36). In a phase I clinical trial, eight patients with refractory AML or high risk MDS received T-cells transduced with a WT1-specific TCR-gene using a retroviral vector encoding small interfering RNAs for endogenous TCR genes (37). The safety profile reported was favourable and TCR T-cells had prolonged persistence, yet clinical benefit was limited.

Despite all these efforts, immunotherapy in AML has yet to provide a curative solution for most AML patients. Antibody-based therapies have shown some efficacy, but only GO has shown sufficient efficacy to be incorporated into standard treatment in clinical practice for certain patients. The more elaborate Ab-based technologies including BITEs, BIKEs and DARTs, and also vaccines have attempted to harness the patient’s own immune system to battle specific leukemic antigen targets. However, these technologies rely on the ability of the native immune effector cells to competently eliminate the malignant cells. In reality, the leukemic cells are able to evade the native immune system, and therefore enhanced function of immune effector cells is required. TCR T-cells have been a step in the right direction, though antigen targeting might be limited due to MHC-dependent recognition. Therefore, novel immunotherapies with the capability of robust immune reactivity are needed.

## Current State of CAR T in AML

Adoptive T cell transfer constitutes an innovative strategy of harnessing effector immune cells to battle malignancy. Chimeric antigen receptor (CAR) T cells contain fusion proteins composed of an antigen-binding extracellular portion that is usually derived from an antibody and an intracellular signalling portion derived from T cell signalling proteins, with additional costimulatory domains from receptors such as CD28, OX40, or CD137 (38).

Autologous CD19 CAR T therapy has been introduced into the field of hemato-oncology with the demonstration of ground-breaking results for patients with R/R B Cell lymphoma (39) and B cell - acute lymphoblastic leukemia (ALL) (40), with response rates of 64% and 81% in the initial reports, respectively. Following the success in B cell malignancies, efforts have been made to engineer CAR products that could target AML, yet this has been hindered by several obstacles.

### Challenges With CAR Therapy in AML

#### Finding the Right Antigen Target

In order to manufacture a potent yet safe CAR, a suitable antigen would need to be expressed on all or most of the leukemic cells, with minimal expression on normal hematopoietic tissue (to avoid aplasia) or other organs (to avoid on target/off tumor toxicity). Furthermore, the ideal Ag should be important for tumor function and should not be expressed on activated T cells, so that CAR efficacy would not be hampered. This is further complicated by the inherent heterogeneity of AML, with coexistent clonal diversity and leukemic stem cells (LSCs). The holy grail of AML immunotherapy is to find the perfect antigen, one that is expressed solely by the AML cell, including the LSC, and that is a driver for AML proliferation. Numerous antigens are being investigated in the quest for to find the ideal AML target. By far, the majority of CAR T or NK cell antigens are cell surface antigens, which are commonly expressed by normal HSCs. Targeting intracellular antigens with CAR T or NK cells is a much more laborious task that involves expression of the intracellular antigen, or segments of the antigen, on the cell surface.

##### CD33 and CD123

Both these antigens are frequently expressed on AML blasts, and are also found on normal hematopoietic cells of myeloid lineage, raising concerns of prolonged myelosuppression as a result of an on target/off tumor effect. Innovative strategies under development to circumvent these obstacles are described in later sections.

A report of one patient who received autologous anti-CD33 CAR Ts for refractory AML has been published (41). The patient experienced grade 4 fever and chills after CAR T infusion, as well as worsening pancytopenia. Despite initial decrease in BM blasts, the patient died 13 weeks after CAR T infusion due to disease progression. The CARs were detected in the peripheral blood concomitantly with progression of CD33 positive leukemic blasts, giving rise to several possible explanations for treatment failure, including inability of the CAR to target all present CD33 isoforms.

CD123, a hallmark marker of blastic plasmacytoid dendritic cell neoplasm (BPDCN) is also abundantly expressed on AML blasts and LSCs. An initial report of the phase I trial with autologous CD123 directed CAR T with a CD28 co-stimulatory domain described the experience with the first 7 patients treated (6 AML and 1 BPDCN with R/R disease) (42). The treatment was safe without significant myelotoxicity, and no CRS grade 3 or higher was noted. Efficacy results were promising, with 5 of 6 AML patients responding, including one CR and one morphologic leukemic-free state.

##### CD38

CD38 is expressed on most AML blast cells and MM plasma cells but not on healthy hematopoietic stem cells. Cui et al. reported their experience in administrating anti CD38 CAR Ts in six patients with relapsed AML after alloHCT. The infused CAR Ts were autologous in four and donor-derived in the remaining two. Four patients achieved CR/Cri, and three patients remained alive and in CR at six months, including one patient who relapsed and achieved renewed CR after a second infusion of CAR Ts (43).

##### FLT3

FLT3 is a transmembrane receptor tyrosine kinase which has a vital role in maintaining normal hematopoietic stem cell (HSC) and progenitor cell function, including proliferation and differentiation. FLT3 mutations occur in approximately one third of AML patients and confer a worse prognosis (44). Wang et al. described potent *in vitro* cytotoxicity of FLT3 CAR Ts in AML cell lines, especially in cells harbouring the FLT3-ITD mutation (45). Surprisingly, normal CD34+ HSC colony formation was not inhibited by the CARs. Administration of the anti-FLT3 CAR also prolonged survival of mice in a human FLT3+ AML xenograft mouse model.

##### Lewis Y (LeY)

LeY is a difucosylated carbohydrate antigen expressed on various solid and hematologic malignancies, including AML. It has limited expression on normal tissue. Early phase 1 data published by Ritchie et al. provided “proof of concept” for CAR T in AML (46). Second generation LeY CAR Ts with CD28 co-stimulation were administered to four AML patients, one with active disease and three with cytogenetic positive MRD. No grade 3-4 adverse events were reported. In the patient with active leukemia, a temporary reduction in peripheral blood blast cells was observed. One other patient achieved a cytogenetic remission, while the other two patients had stable disease. CAR trafficking to the BM was documented using In111-labeling, and the CAR persisted in peripheral blood for up to 10 months. Of note, the conditioning regimen included Fludarabine and high dose cytarabine (2gm/m2 for 5 days), although the abnormal cytogenetic clone was still observed in three of four patients after this conditioning and prior to CAR administration (46).

##### NKG2D Ligand

Expressed in a variety of hematologic and solid tumors and has limited expression on healthy tissues. Therefore, it has been examined as a target for autologous CAR T therapy in a phase I study (47). Twelve patients were enrolled, including 7 with AML (the remainder with multiple myeloma). The CARs were administered without lymphodepleting conditioning. The toxicity profile was favourable, yet none of the patients achieved an objective response per protocol. One patient did achieve stability in some laboratory and clinical parameters for several months without further treatment.

##### CD117

CD117 (c-kit), the cognate receptor for stem cell factor, is expressed on most AML blasts as well as healthy hematopoietic stem and progenitor cells. CAR Ts targeting CD117 with *in vitro* and *in vivo* preclinical efficacy in elimination of leukemic cells have been constructed (48). In order to avoid sustained myloablation, CAR Ts co-expressing RQR8 were depleted *in vivo* using rituximab administration and ATG. This could potentially enable using anti-CD117 CAR Ts as part of conditioning regimens prior to alloHCT.

The pursuit of novel CAR targets is the subject of rigorous investigation, and novel antigens have been recently reported. For instance, preclinical data suggests Siglec-6 could also be a suitable CAR T target in AML, since it is widely expressed on AML blasts as well as LSC, yet not on HSC or hematopoietic progenitor cells, unlike other Siglec-family members such as Siglec-3 (i.e. CD33) (49).

An innovative strategy has been developed to target the PR1/HLA-A2 complex on AML cells using peripheral blood and umbilical cord–derived TCR-like h8F4-CAR Ts. PR1 is present in primary azurophilic granules of neutrophils, but also aberrantly overexpressed on HLA-A2 in myeloid blasts. These CAR-T cells have been shown to effectively eliminate AML blasts *in vitro*, providing proof of concept of the ability of CARs to target intracellular peptides bound to a surface MHC molecule (50). This expands the pool of possible targetable antigens beyond the traditionally researched extracellular proteins.

### Battling Tumor Resistance Mechanisms

Despite the successful clinical experience with CD19-directed CAR Ts in B-Cell lymphoid malignancies, long term follow up has revealed that up to 50% of patients have disease relapse (39, 40, 51).

Learning from experience attained from use of CAR T in lymphoid malignancies, two major patterns of relapse have been described. The first, *relapse with antigen-positive disease*, typically seen early within several months of CAR T infusion with B-ALL. In one report, all nine adult ALL patients with MRD positive CR after anti-CD19 CAR T relapsed with CD19 positive leukemia (51). This pattern often coincides with lack of CAR T persistence or B Cell aplasia. This issue could be addressed by optimization of the CAR T design, including co-stimulatory domains, recurrent CAR infusions and enhancing efficacy *via* combination therapies to prolong substrate persistence. The second pattern is *relapse with antigen loss*, typically seen later during follow up, whereby tumor cells undergo either complete antigen loss, antigen modulation or reduced expression on tumor cells. In a phase I trial of children and young adults with ALL, 14% of those who achieved CR following CAR T infusion, developed antigen negative relapse (52). Clonal heterogeneity, especially worrisome in AML, could also predispose to refractoriness to CARs *via* selection of antigen-negative clones.

Using multiple Ag targets in parallel or sequential manner could potentially abrogate antigen modulation. In a report by Perna et al., proteomics and transcriptomics were utilized to discover potential antigens for CAR targeting. Combinatorial target search yielded several potential CAR pairs, including CD33+ADGRE2, CLEC12A+CCR1, CD33+CD70, all three positively stained more than 97% of cells in AML samples and less than 5% of normal HSCs and T cells (53). Using a detailed protein expression profile based on flow cytometry of AML and normal bone marrow, Haubner et al. identified CD33/TIM3 or CLL1/TIM3 combinations as potential effective and safe CAR target pairs (54).

Early clinical results of a dual CLL1-CD33 CAR T composed of two constructs connected by a cleavable linker (P2A) for patients with R/R AML have been reported. Eight patients received an autologous product, and a ninth received from an HLA matched sibling donor. CRS occurred in 8 of the 9 patients, including 2 grade 3 events. Neurotoxicity occurred in 4 patients, including 3 grade 3. All CRS and neurotoxicity resolved. Other toxicity included grade 4 pancytopenia in all patients. 7/9 of patients achieved MRD negative at 4 weeks after CAR T infusion. Six of these went on to undergo allogeneic HSCT, and 5 of these successfully engrafted (55).

Combining CARs with other treatment modalities could potentiate the CAR effect. For instance, in B-ALL, CPI have been shown to prolong persistence of CAR Ts (56). The addition of atezolizumab to axi-cel for patients with R/R DLBCL yielded a higher CAR T-cell expansion in the phase 1 of ZUMA-6 compared to results obtained in ZUMA-1. 90% of the 12 patients responded, including 60% CR. Grade 3 or higher CRS and neurotoxicity were reported in 25% and 50% of patients, respectively (57). Phase II of ZUMA-6 is currently ongoing. In AML, preclinical data suggest that the addition of crenolanib to FLT3-directed CAR T has synergistic effect in patients with the high risk FLT3-mutation (58).

Improved understanding of clonal kinetics of infused CAR T cells could provide insight into possible improvements in tumor control. Infused CAR T cells are polyclonal, and after infusion they undergo selective clonal expansion (59). Sheih et al. described the dynamic gene expression profile of infused CD8+ cells. Early after infusion, they observed that cells show an activated CD8+ transcriptional signature, whereas later on the activation transcriptional progressively declined, though without increased expression of genes associated with T-cell exhaustion (59).

Some evidence has suggested differential efficacy of virally transduced CAR T cells depending on the genomic site of integration. For instance, in depth analysis of CAR T populations in a patient with chronic lymphocytic leukaemia who achieved complete response after treatment with anti-CD19 CAR T revealed that at peak response most cells originated form a single TET2-disrupted clone (60). Further *in vitro* studies showed increased expansion and a distinct cytokine release pattern which suggest improved efficacy of TET2-disrupted CAR T cells.

## Resistance Mechanisms in AML

Mutations in certain genes have been shown to confer resistance of the AML blasts. For instance, FLT3 mutations, found in approximately 40% of normal karyotype AML cases, The FLT3-ITD aberration has traditionally been associated with worse outcomes with chemotherapy. Leukemic cells also possess inherent functional resistance mechanisms, such as drug efflux pumps and cell cycle properties (61). Furthermore, selective pressure imposed by chemotherapy or biological agents has been shown to give rise to resistant leukemic clones (62). AML can evade immunotherapy through up-regulation of T-cell inhibitory ligands, anti-apoptotic proteins and regulatory T-cell population, and by inhibition of T-cell immune synapse formation and NK cytolytic function (63). It remains to be seen whether CAR therapy could provide an adequate solution to overcome such resistance mechanisms.

### Managing CAR Toxicity

Several aspects of CAR T related toxicity could potentially be more troublesome in AML patients. Cytopenias and infectious complications could potentially be more prominent than those previously reported in clinical trials with CAR T cells in lymphomas, owing to older age of AML patients, previous intensive therapies and treatment related morbidity, especially when using CARs in the R/R setting. Furthermore, as previously mentioned, some antigens targeted by the CARs, such as CD33 and CD123, could potentially worsen myelosuppression due to expression on HSC and hematopoietic progenitors (41, 42).

A technical approach being developed to reduce CAR toxicity is an “off switch” which limits CAR persistence. One option is using a “suicide gene”, such as herpes simplex virus-thymidine kinase (HSV-tk), which enables selective elimination of expressing cells when the appropriate signal is delivered. Preclinical studies incorporating the HSV-tk suicide gene into a CD44v6-directed CAR Ts in AML and MM cells showed successful CAR inhibition upon exposure to ganciclovir (64). Other technologies to enable toxicity modulation through gene editing include CRISPR (65) and TALEN (66), as described later in this review.

Targeting multiple antigen targets could reduce potential on target/off tumor CAR T toxicity, by enhancing leukemic specificity (53). A novel approach to mitigate hematological toxicity of CD33-targeted CAR T therapy has been proposed, whereby alloHCT of hematopoietic progenitors lacking CD33 using gene editing could facilitate subsequent safe infusion of anti-CD33 CAR Ts. Preliminary preclinical data have suggested feasibility of this strategy (67).

## The Need for Speed in AML: Allogeneic CARs

Due to the aggressive nature of the disease, one of the challenges of potential CAR therapy for AML is delivering the cells in a timely manner. With autologous CAR T cells, disease progression during the manufacturing turnover time (up to three weeks in the case of CD19 autologous CAR T cells) is a serious concern. This is further complicated in AML, where prior immunosuppressive therapies can make effector cell collection and CAR manufacturing especially troublesome. Use of allogeneic CARs could help overcome these obstacles, providing off-the shelf ready-to-use products from healthy donors.

Autologous CAR T cell products also have inherent variability in the T cell population. In a clinical trial of anti-BCMA autologous CAR T cell treatment for multiple myeloma, the infused CD4:CD8 T cell ratio and frequency of CD45RO-CD27+CD8+ T cells correlated with CAR T expansion as well as clinical responses (68). A comprehensive molecular landscape of autologously derived CAR T cells was recently published by Chen et al. Transcriptomic analysis of the anti-CD19 CAR T product in 71 pediatric patients with B cell malignancies was complemented by single cell analysis of six patients (69). Authors found that the TCF7 gene was vital in maintaining effector cell persistence, whereas IRF7, a regulator of the IFN response pathway, was associated with poor CAR T cell persistence.

Different effector cells that could be used for these universal CARs include αβT cells/γδT cells/NK cells. Nonetheless, infusion of allogeneic activated immune effector cells does bear the potential for causing graft versus host disease (GVHD). Several mechanisms to avoid GVHD have been investigated. One option would be to neutralize the TCR. This has been investigated by removal of TCR using CRISPR gene–editing. TCR and HLA class I double deficient T cells displayed reduced alloreactivity and did not cause GVHD, while preserving antitumoral potency *in vitro* and in murine models (65). TCRα gene disruption *via* transcription activator-like effector nucleases (TALEN) gene editing has also shown preclinical promise (66). Other options to avoid GVHD include selection of specific effector T cells subsets for the CAR construct that are less likely to incite GVHD, such as memory T cells or γδT cells (70), or rituximab-mediated CAR depletion (71). At least to date, limited clinical experience has not substantiated these concerns for excessive GVHD in the setting of allogeneic CARs. In a report of 20 patients who received allogeneic CD19 targeting CAR Ts for B cell malignancies relapsing after alloHCT, none developed new GVHD, whereas 8 of the 20 patients responded (including 6 CRs) (72).

**Figure 1:**
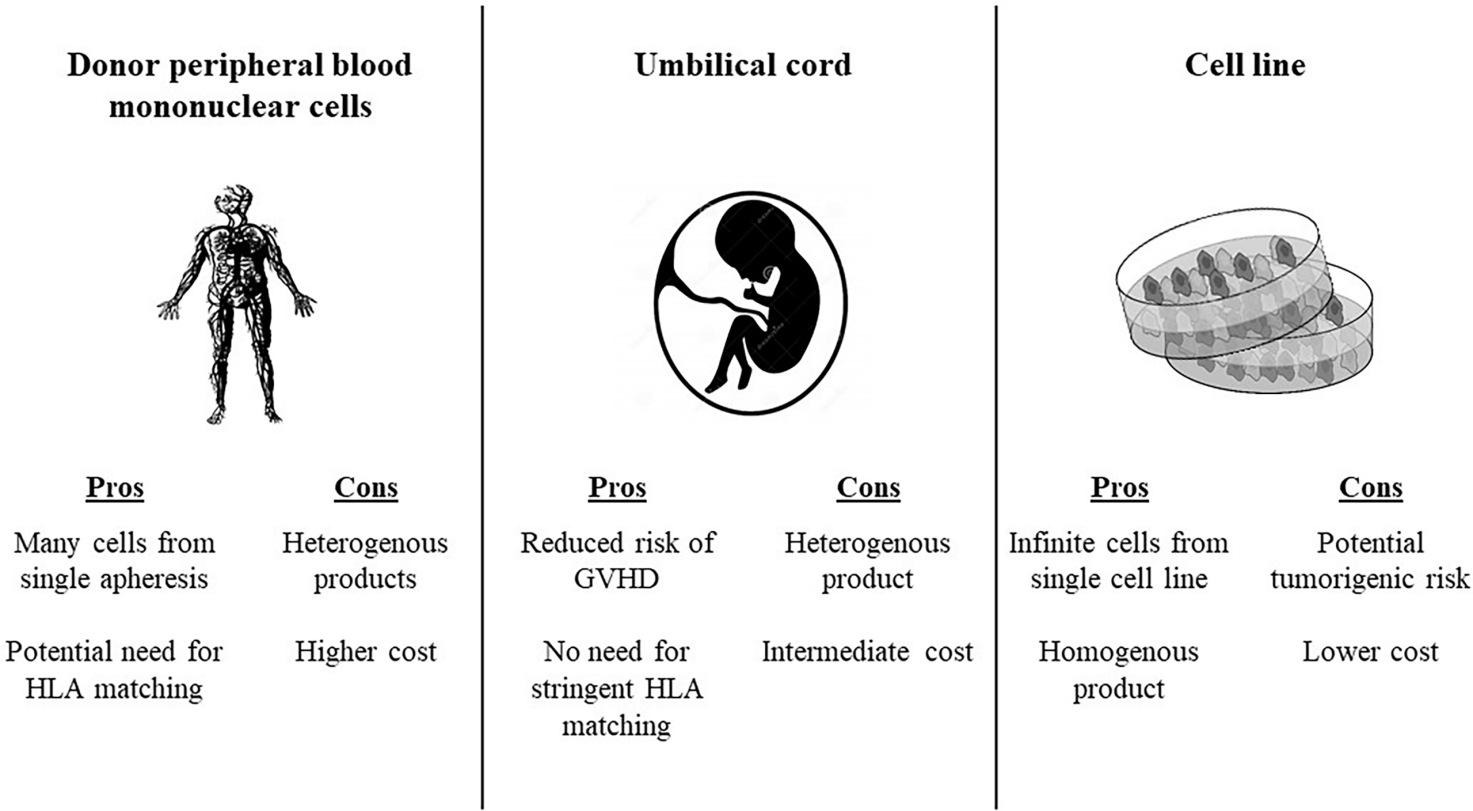
Potential sources for allogeneic CAR products. HLA, human leukocyte antigen; GVHD, graft versus host disease.

There are several potential sources of allogeneic effector cells to for the CAR construct (73) (**Figure 1**). Using Healthy donor peripheral blood mononuclear cells enables production of many vials form a single apheresis product, and offers potential banking of different HLA typed products to facilitate the option of HLA typing. Using umbilical cord blood as a source for effector cells reduces the risk of GVHD, and consequently abrogates the need for stringent HLA-matching between the CAR source and the recipient. Placenta-derived T or NK cells have a unique HLA pattern and could potentially be incorporated into CAR constructs as well. Induced pluripotent stem cells (iPSCs) offer the potential of a renewable and homogenous source of effector CAR cells, since these are generated from a single clonal engineered pluripotent cell line (74). Virus-specific T cells have been used as a platform for CAR expression. An early clinical trial in neuroblastoma showed that human cytotoxic T lymphocytes (CTLs) loaded with a synthetic CAR directed against a tumor associated antigen had prolonged persistence and had anti-tumor activity (75). Gene editing has been used to avoid other potential drawbacks of allogeneic CAR Ts. For instance, CRISPR/Cas9 gene editing has enabled construction of anti-CD7 allogeneic CAR Ts that lack both CD7 and T cell receptor alpha chain (TRAC) expression. These fratricide-resistant allo CARs have *in vivo* and *in vitro* efficacy against T cell acute lymphoblastic leukemia (T-ALL). CD7 is expressed on approximately one quarter of AML blasts and is considered a marker of leukemic stem cells, thus could be a potential target for allogeneic CAR T in AML (76)

### Early Clinical Experience With Allogeneic CARs

There have been few reports of clinical experience with allogeneic CAR T for AML in the literature. As previously mentioned, two of the six patients described in the report by Cui et al. who received a CD38 CAR T, received a donor-derived CAR construct (43) In one of the patients who received a dual CLL1 CD33 CAR T, the donor was a HLA matched sibling (55). Two Phase 1 clinical trials (for AML NCT03190278, and BPDCN NCT03203369) with the unrelated-donor derived allogeneic anti-CD123 CAR Ts uCD123 were suspended after the first recruited 78-year-old patient with BPDCN died because of severe CRS and capillary leak syndrome. The first patient in the AML trial with this agent also experienced severe CRS and capillary leak syndrome, though his condition later improved. This trial has resumed with modifications in CAR T dose and age eligibility criteria (see **Table 1**).

**Table 1:** Ongoing clinical trials with allogeneic CARs in AML.

Antigen target	CAR source and effectors	Phase	Key eligibility criteria	Lympho-depletion	ClinicalTrials.gov ID	Company/Institute	Trial status
CD33, CD123 or CLL-1 (multiple CAR Ts)	Auto/donor T Cells	1/2	Age 6 Months to 75 Years	NA	NCT04010877	Shenzhen Geno-immune Medical Institute, Guangdong, China	Recruiting
CD123	Donor T Cells	1	Relapse after allo-HCT	CyFlu	NCT03114670	Affiliated Hospital to Academy of Military Medical Sciences, Beijing, China	NA
CD33	Off the shelf NK Cells	1	Not eligible for allo-HCT	CyFlu	NCT05008575	Xinqiao Hospital Chongqing, China	Not yet recruiting
CD33	Auto/donor T cells	1/2	- CR2 or CR3 and not eligible for allo-HCT - Less than 1 year between last chemotherapy and progression - Relapsed after prior autologous or allogenic HCT	NA	NCT01864902	Affiliated Hospital of Changzhi Medical College Beijing, China	NA
CD33	Off the shelf NK cells (NK92)	1/2	- most recent progression free interval < 1 year - AML in CR2 or CR3 and not eligible for allo-HCT	CyFlu	NCT02944162	PersonGen BioTherapeutics (Suzhou) Co., Ltd. Jiangsu, China	NA
CD123	Auto/donor (relatd or unrelated) T cells	1	- 12 years and older - AML/BPDCN - R/R or high risk for recurrence	CyFlu	NCT02159495	City of Hope Medical Center California, USA	Recruiting
CD123	Allogeneic T Cells	1	- 18 to 65 years	NA	NCT03190278	Cellectis S.A. (5 sites in USA)	Recruiting
CD123	allogenic or autologous T Cells	1/2	- R/R AML, ineligible for allo-HCT - 14-75 years	CyFlu	NCT03556982	307 Hospital of PLA Beijing, China	NA
IM73	Donor T Cells	1	- Age ≥ 18 years - R/R AML	CyFlu	NCT04766840	Peking University People’s Hospital Peking, China	Not yet recruiting
CD7*	Auto/donor T Cells	1/2	- Age 6 Months to 75 years	NA	NCT04033302	Shenzhen Geno-immune Medical Institute, Guangdong, China	Recruiting
CD7	Off the shelf NK Cells	1/2	- Age ≥ 18 years - CD7 positive R/R Leukemia and Lymphoma	NA	NCT02742727	PersonGen BioTherapeutics (Suzhou) Co., Ltd., Jiangsu, China	NA
CD123	Allogeneic T Cells (Donor)?	1	- Age ≥ 18 years - AML, B-ALL or BPDCN	CyFlu	NCT04230265	Cellex Patient Treatment GmbH (5 sites in Germany)	Recruiting
CD33	Allogeneic T Cells	1	- Age ≥ 50 years	NA	NCT02799680	Affiliated Hospital of Academy of Military Medical Sciences Beijing, China; Chinese PLA General Hospital Beijing, Beijing, China	NA

CyFlu, cyclophosphamide fludarabine; NA, data not available; Allo-HCT, allogeneic hematopoietic cell transplantation.

*Combined with alternative targeting CAR-T cells.

Other concerns have been raised with the use of allogeneic CARs, including risk of potential genomic abnormalities. These could be promoted by genetic manipulations used to modify the infused products, for instance with CRISPR or TALEN technologies for TCR removal (65, 66). Recently, ALPHA-2, a phase I study with allogeneic CAR Ts for adults with large B-cell lymphoma (NCT04416984) has been halted by the FDA due to a chromosomal abnormality noted in ALLO-501A CAR T cells found in the bone marrow of a trial participant with pancytopenia (77). The clinical significance of this abnormality is unclear at this time.

## NK Cells to the Rescue? From Adoptive NK cells to Allogeneic CAR NKs

NK cells are highly cytotoxic effector immune cells, with an ability to target virally infected or cancerous cells (78). In the context of allogeneic CAR constructs, these cells have the added appeal of not causing GVHD (79). Clinical results with adoptive NK cells have shown mixed results, perhaps in part due to differences in methodology and patient selection.

As early as 2005, Miller et al. published their experience with infusion of haploidentical, related-donor NK-cells with IL2 priming in 43 adult patients with various cancers (19 of them with active poor-prognosis AML) (80). Patients received either low or high intensity conditioning regimens prior to NK infusion. Five of the AML patients achieved remission, however toxicity was substantial, including pancytopenia, fever, pleural effusions, hypoxemia, and constitutional symptoms, as well as prolonged hospitalization Expansion of donor NK cells occurred only after the high intensity regimen. In efforts to improve response, adoptive transfer of haplo-identical killer immunoglobulin-like receptor–human leukocyte antigen (KIR-HLA) mismatched NK cells have been used in children with AML, with reports demonstrating good tolerance but mixed results with respect to efficacy (81, 82).

Anti-leukemic activity of cytokine-preactivated allogeneic NK cells in AML has been previously shown to be feasible with preliminary signs of efficacy, generating response in five out of nine evaluable patients with R/R AML, including four CRs (83). The NK-92 cell-line (aNK) is a human interleukin (IL)-2-dependent NK cell line with expression of activating receptors, but without most of the KIRs. After preclinical *in vivo* and *in vitro* data revealing high levels of anti-leukemic cytotoxic activity, clinical evaluation of these “off the shelf” allogeneic aNK cells was performed in a phase I study (74). Seven patients with R/R AML received aNK infusions. Therapy was generally safe, with no grade 3-4 toxicities noted and only one patient experienced grade 2 fever and chills following each aNK cell infusion. However, efficacy was limited, with one patient experiencing reduction in blast count and two having a stable blast percentage at 21 days post first aNK infusion.

Umbilical cord blood-derived NK cells have been used as another form of allogeneic NK therapy, for instance in combination with autologous transplant in patients with multiple myeloma (84). Anti-CD19 CAR NKs have been constructed using retroviral vector modification incorporating the genes for CAR-CD19, IL-15 and inducible caspase-9-based suicide gene (iC9), which demonstrated both *in vivo* and ex vivo as well as clinical efficacy against B-cell malignancies (79, 85).

Human iPSC are being investigated as another potential source for NK CAR cells. In an ovarian cancer xenograft model, iPSC CAR NK cells inhibited tumor growth and prolonged survival (86). NK signaling and antigen-induced cytotoxicity were optimized by building a construct containing transmembrane NKG2D and intracellular 2B4 domains. Due to the pluripotent undifferentiated nature of the iPSC, a concern of potential tumorigenic risk has been raised (87).

## Conclusion and Future Strategies

The path to successful incorporation of CAR T therapy into widespread use for AML patients is riddled with challenges. These include finding an appropriate antigen target, having the CAR manufactured and available in a timely manner and avoiding excess toxicity to the patients as well as disease resistance. Designing allogeneic “off the shelf” CAR construct could potentially resolve several of these issues, while creating other challenges, such as possible increased risk of GVHD due to HLA-mismatch between the donor and recipient. Research efforts are striving to overcome these issues. Ongoing clinical trials using allogeneic CARs are listed in **Table 1**.

One strategy with potential for implementing CAR Ts in AML is targeting leukemia associated (as opposed to leukemia specific) antigens. This would allow more robust elimination of leukemic cells. Since these antigens are expressed also on normal hematopoietic cells, targeting these antigens could theoretically cause prolonged myelosuppression. As opposed to patients with solid tumors, for patients with AML, alloHCT is standard therapy and could therefore be used if cytopenia persists. Targeting leukemia associated antigen, such as PR1 and WT1, with immunotherapy has been shown to be efficacious and safe in clinical AML studies (34, 88).

In B cell malignancies, autologous CD19-directed CARs have provided long lasting remissions without the need for additional treatment (39, 40, 51). However, disease biology and clonal selection in AML might dictate the need for sequential therapy due to a limited duration of remission conferred by cellular therapy in this disease. Indeed, in several studies, both autologous (55) and allogeneic (89) CARs have been used as bridging therapy to allogeneic HSCT in acute leukemia. We are at the beginning of the road for CAR T therapy in AML, and time will tell whether this modality will provide the much-needed stepping stone for improved patient outcomes.

## Author Contributions

OP contributed to development, writing, and final review of the article. MD contributed to writing and final review of the article. GA contributed to writing and final review of the article. DM contributed to writing and final review of the article. ND contributed to writing and final review of the article. FR contributed to writing and final review of the article. KR contributed to writing and final review of the article. ES contributed to writing and final review of the article. PK contributed to development, writing, and final review of the article. All authors contributed to the article and approved the submitted version.

## Conflict of Interest

KR: KR and The University of Texas MD Anderson Cancer Center have an institutional financial conflict of interest with Affimed GmbH and Takeda Pharmaceutical. KR participates on the Scientific Advisory Board for GemoAb, AvengeBio, Virogin, GSK, Bayer, Navan Tech, and Caribou. FR: Advisory board member and honoraria from BMS. ND has received research funding from Daiichi-Sankyo, Bristol-Myers Squibb, Pfizer, Gilead, Sevier, Genentech, Astellas, Daiichi-Sankyo, Abbvie, Hanmi, Trovagene, FATE therapeutics, Amgen, Novimmune, Glycomimetics, Trillium, and ImmunoGen and has served in a consulting or advisory role for Daiichi-Sankyo, Bristol-Myers Squibb, Arog, Pfizer, Novartis, Jazz, Celgene, AbbVie, Astellas, Genentech, Immunogen, Servier, Syndax, Trillium, Gilead, Amgen, Shattuck labs, and Agios. ES: Consultant on Scientific Advisory Boards: Novartis, Magenta, Adaptimmune, Axio, Navan; License Agreements or Patents: Takeda, Affimed; Honorarium: Magenta, Novartis, Bayer HealthCare Pharmaceuticals. PK: Consultant on Scientific Advisory Boards: Novartis, Jazz, Pfizer, Kite, Jasper.

The remaining authors declare that the research was conducted in the absence of any commercial or financial relationships that could be construed as a potential conflict of interest.

## Publisher’s Note

All claims expressed in this article are solely those of the authors and do not necessarily represent those of their affiliated organizations, or those of the publisher, the editors and the reviewers. Any product that may be evaluated in this article, or claim that may be made by its manufacturer, is not guaranteed or endorsed by the publisher.
